# Utilization of genetic data can improve the prediction of type 2 diabetes incidence in a Swedish cohort

**DOI:** 10.1371/journal.pone.0180180

**Published:** 2017-07-12

**Authors:** Hadi Zarkoob, Sarah Lewinsky, Peter Almgren, Olle Melander, Hossein Fakhrai-Rad

**Affiliations:** 1 BaseHealth Inc., Sunnyvale, California, United States of America; 2 Department of Clinical Sciences, Lund University, Malmö, Sweden; McMaster University, CANADA

## Abstract

The aim of this study was to measure the impact of genetic data in improving the prediction of type 2 diabetes (T2D) in the Malmö Diet and Cancer Study cohort. The current study was performed in 3,426 Swedish individuals and utilizes of a set of genetic and environmental risk data. We first validated our environmental risk model by comparing it to both the Finnish Diabetes Risk Score and the T2D risk model derived from the Framingham Offspring Study. The area under the curve (AUC) for our environmental model was 0.72 [95% CI, 0.69–0.74], which was significantly better than both the Finnish (0.64 [95% CI, 0.61–0.66], p-value < 1 x 10^−4^) and Framingham (0.69 [95% CI, 0.66–0.71], p-value = 0.0017) risk scores. We then verified that the genetic data has a statistically significant positive correlation with incidence of T2D in the studied population. We also verified that adding genetic data slightly but statistically increased the AUC of a model based only on environmental risk factors (RFs, AUC shift +1.0% from 0.72 to 0.73, p-value = 0.042). To study the dependence of the results on the environmental RFs, we divided the population into two equally sized risk groups based only on their environmental risk and repeated the same analysis within each subpopulation. While there is a statistically significant positive correlation between the genetic data and incidence of T2D in both environmental risk categories, the positive shift in the AUC remains statistically significant only in the category with the lower environmental risk. These results demonstrate that genetic data can be used to increase the accuracy of T2D prediction. Also, the data suggests that genetic data is more valuable in improving T2D prediction in populations with lower environmental risk. This suggests that the impact of genetic data depends on the environmental risk of the studied population and thus genetic association studies should be performed in light of the underlying environmental risk of the population.

## Introduction

Type 2 diabetes, the most common form of diabetes, is a rising healthcare problem worldwide. The number of people affected with type 2 diabetes has risen significantly over the past 30 years. The global prevalence of diabetes among adults over 18 years of age has increased from 4.7% in 1980 to 8.5% in 2014. This resulted in 1.5 million deaths due to diabetes, making it the eighth leading cause of death [1].

As a multifactorial disease, the risk of developing T2D is determined by different types of RFs. Behavioral and clinical RFs (together called environmental RFs in this article), as well as genetic factors contribute to the development of T2D [2–3]. Numerous epidemiological studies have reported associations between behavioral and lifestyle RFs, such as diet, smoking, physical activity and BMI, as well as blood and phenotypic markers, such as triglycerides, gender and the development of T2D. Subsets of these RFs have been used to create phenotypic diabetes risk scores such as the risk score derived from the Framingham Offspring Study [4] and the Finnish Diabetes Risk Score [5]. However, these risk scores do not incorporate genetic data. For decades, scientists have been studying how variations in the genome contribute to variations in disease risk. The association between genetics and the development of T2D has been repeatedly reported through linkage analysis, twin studies and Genome Wide Association Studies (GWAS). However, the genetic markers identified thus far have all shown low penetrance. Therefore, to accurately assess an individual’s risk of T2D one needs to consider all of these factors. The risk assessments in this study are based on a multifactorial risk assessment engine, named BaseHealth^™^ Risk Engine, which integrates the genetic and environmental RFs to perform a disease risk assessment [6–7].

One of the limitations in the design of genetic studies, specifically GWAS, is the lack of environmental risk stratification. In most studies a group of people who have been diagnosed with T2D (cases) are assessed against a group of people who have not yet been diagnosed with T2D (controls). However, there is typically no detailed risk stratification available within each of these groups. Within a group of people with T2D there will likely be different levels of environmental and genetic risk. The same applies to a group of people without T2D. Some will likely have higher risk of developing T2D in the future due to either genetic or environmental RFs. Not accounting for detailed stratification of environmental RFs when evaluating the effect of genetic risk can affect the scope of interfacing between the environmental and genetic RF.

The purpose of this study is three-fold: (i) to investigate whether the incidence of T2D in a Swedish cohort varies when stratifying the population by level of genetic and environmental risk, (ii) to determine whether the impact of genetic predisposition varies across different environmental risk groups and (iii) to determine whether adding genetic data improves the sensitivity and specificity of an environmental type 2 diabetes risk assessment.

## Materials and methods

### Risk assessment engine (RAE)

The medical information and statistical data placed within the risk assessment engine from BaseHealth^™^ are chosen after stringent filtration. The inclusion criteria for the environmental [8–23] and genetic [24–52] studies include quality of the data (study design, sample size and statistical methods), source of data, documented reproducibility, and correlation to either progression or prevention of type 2 diabetes. Reports based on expert consensus, guidelines, and majority practice patterns were not included.

**Fig 1:**
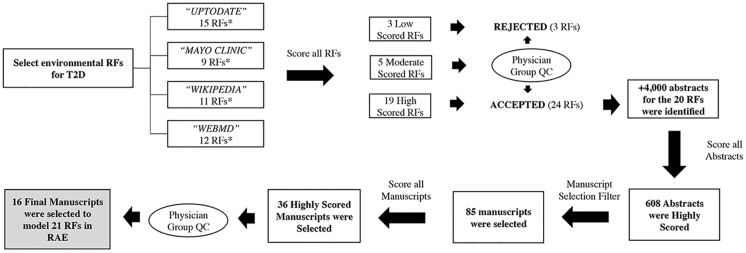
A flow diagram on how environmental RFs for T2D were identified and representative studies were selected to be modeled in the RAE. * Some RFs overlap between data sources.

The process for selecting studies for environmental RFs is illustrated in Fig 1. A list of potential RFs was compiled from four sources (UpToDate, Mayo Clinic, Wikipedia and WebMD). Those RFs were scored based on how many of the sources included them. Based on the resulting scores, RFs were accepted for literature search, rejected or reviewed by the physician group for a final decision. Literature searches of the MEDLINE database were performed for each accepted RF from January 1990 through July 2015 using the MeSH term *diabetes mellitus*, *type 2* plus MeSH terms and keywords for each RF. Searches used for each RF are listed in S1 File. To be considered for inclusion studies must (1) be observational cohort, cross-sectional or case-control studies with type 2 diabetes as an endpoint (2) include a measure of risk such as an odds ratio (OR) with confidence interval (3) have a quantifiable measure of the RF and (4) be of sufficient sample size. Of the 608 abstracts screened, 85 full-text manuscripts met all inclusion criteria after review. These 85 manuscripts were then scored on a number of criteria including sample size, diversity of the population (in terms of age, ethnicity, gender etc.), and employment of a well-adjusted model to narrow down the studies to a handful of representative studies for each RF. A team of physicians reviewed the 36 highest scored manuscripts and selected 16 representative studies covering the 21 environmental RFs that were modeled in the RAE.

**Fig 2:**
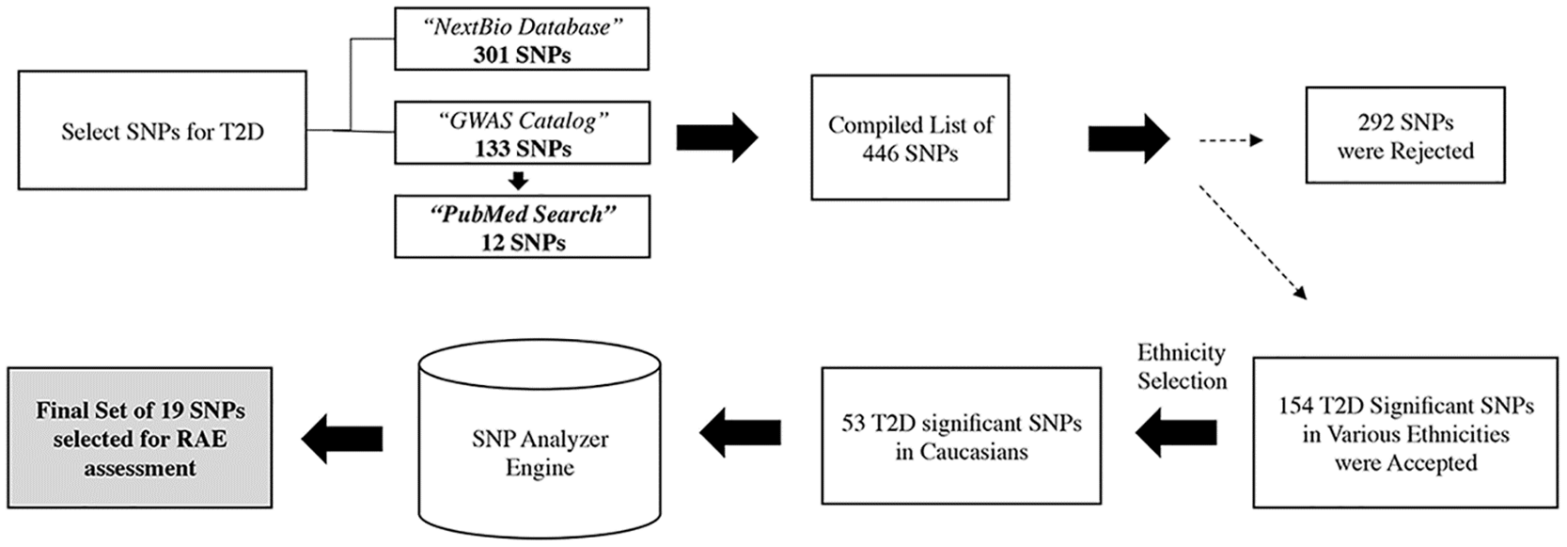
A flow diagram on how SNPs were selected for inclusion in the RAE.

The process for selecting single nucleotide polymorphisms (SNPs) associated with T2D for inclusion in the RAE is illustrated in Fig 2. For studies prior to 2013 a database of GWAS studies was acquired from NextBio Inc. (Now part of Illumina Inc. www.illumina.com]. For studies from January 2013 through July 2015 the NHGRI-EBI Catalog of published genome-wide association studies (available at: www.ebi.ac.uk/gwas) was utilized to identify the studies of interest. In some cases, references from studies found in one of the above databases were also included. Studies were excluded if they didn’t meet a minimal sample size requirement and SNPs were excluded if they did not reach GWAS significance (P-value < 5 × 10^−8^ in most studies). From 29 studies, 154 SNPs from any ethnicity were selected. Because the population for this study was of European descent, SNPs found in other ethnicities were excluded leaving 53 SNPs. These SNPs were run through the SNP Analyzer Engine that scored them based on criteria such as sample size of the study, p-value of the SNP and replication across multiple studies. 25 SNPs that were not replicated across multiple studies were excluded at the end of this step leaving 28 SNPs. These 28 SNPs covered 24 LD blocks in people of European descent. The SNPs in each LD block are ranked based on the scores assigned in the previous step. For 18 out of the 24 LD blocks the highest scoring SNP was available for the majority of individuals in the MDC-CC data (≥ 80% individuals depending on the LD block). For one LD block the highest scoring SNP was not available for any individual because it was not on Illumina’s OmniExpress array used in this study, but the second highest scoring SNP was available for the majority of the individuals (89% individuals). An individual needed to have data for all these 19 LD blocks to be included in the analysis. For five of the LD blocks none of the SNPs were available as they were not available on the array used in this study. As a result, these LD blocks could not be used in the risk calculation. The SNPs were scored in each study they appeared in and the OR was used from the highest scoring study.

**Fig 3:**
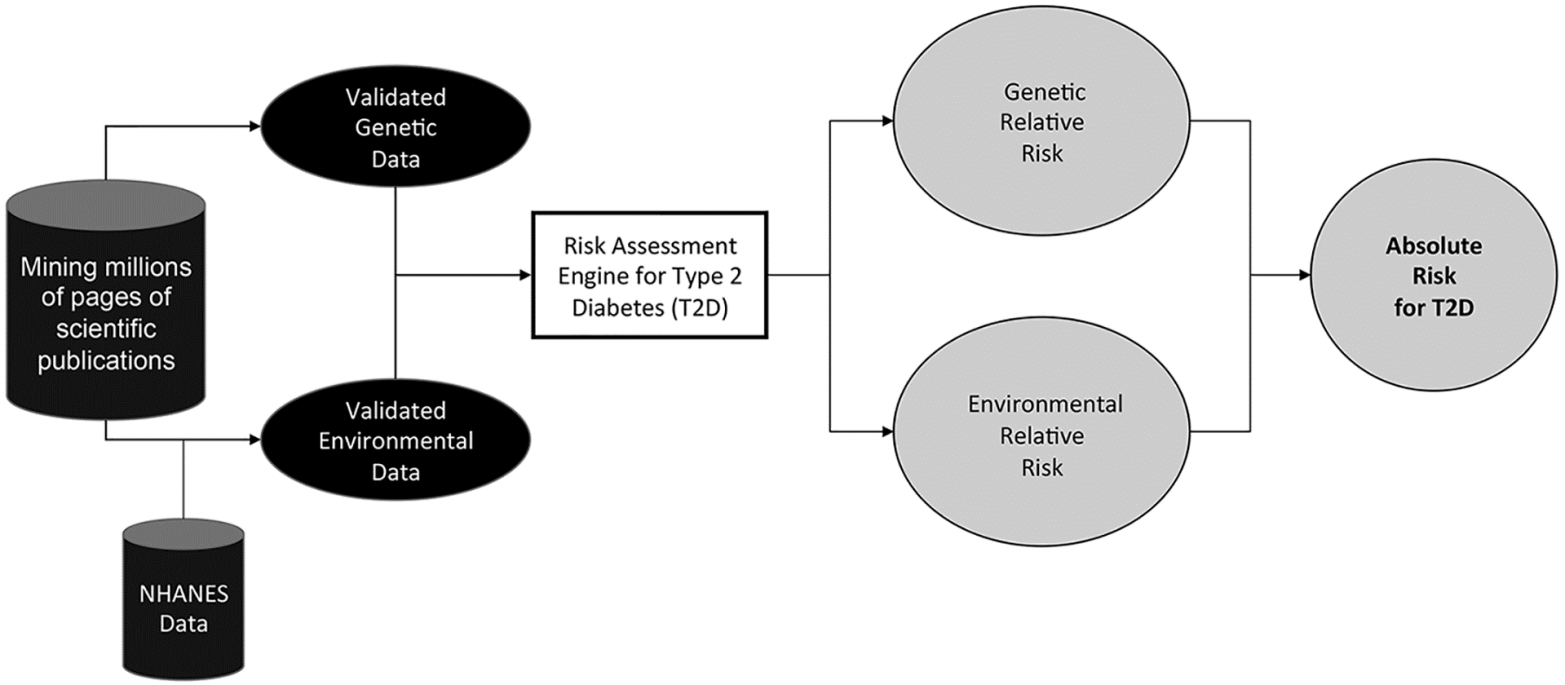
Illustration of the RAE used to assess the risk of type 2 diabetes in the MDC-CC. In this study, T2D relative environmental and genetic risks are used.

The collection of selected studies was used to identify and validate the RFs and their associated effect sizes. Risk is quantified on the engine by an OR. We also considered studies reporting RF effect sizes in the form of a hazard ratio (HR) or a relative risk ratio (RR). In these cases, HRs and RRs are considered an approximation of the true OR. RRs can be a reasonable approximation for ORs (resulting in a relative error of 10% or less) when the prevalence of the disease in the unaffected population is less than or equal to 10% and true ORs are less than or equal to 2 [53]. These criteria are met by type 2 diabetes and the majority of the ORs that are used in the analysis. For a subset of RFs in type 2 diabetes we did not find appropriate data from published studies. In these cases, we built our own statistical models based on the NHANES dataset [8] to calculate the effect sizes of the relevant RFs. ORs obtained for individual RFs are applied to calculate the overall OR at any given age. If an individual was taking blood pressure or lipid-lowering medications at baseline, they were assigned the maximal OR for the corresponding RFs. The health profiles of Malmö Diet and Cancer study participants are submitted to the RAE and overall ORs are calculated for both affected and unaffected individuals as illustrated in Fig 3. S1A Table provides OR ranges for the 21 environmental risk factors used in the study. S1B Table provides allelic odds ratios for the SNPs used by the RAE.

### Design and study population

The Malmö Diet and Cancer study (MDC) is a population-based, prospective study in which inhabitants in Malmö born between 1923 to 1945 (males), or 1923 to 1950 (females), were invited to participate. 28,449 accepted and attended a baseline examination between 1991–1996. A random 50% sample of the participants examined in MDC between 1991–94 (n = 12,445) were invited to also participate in a study on the epidemiology of carotid artery disease, the cardiovascular cohort of the MDC (MDC-CC). 6,094 subjects accepted and underwent a more detailed examination, which has been previously described [54]. Individuals were excluded if they had prevalent T2D at baseline (n = 267) or had missing environmental RF data (n = 1,287). This left 4,540 individuals who had sufficient environmental data and were free of T2D at baseline. All the individuals (n = 497) with censored data were removed so the population includes only individuals with known disease status at the end of the follow-up period. These removed individuals include those who left the study before either receiving a T2D diagnosis or the end of the follow-up period. The environmental and genetic ORs are not significantly different between the excluded 497 individuals and the remaining 4,043 individuals for which the disease status is known at the end of the follow-up period (Two-sided t-test p-values = 0.791 and 0.685 performed on the logarithm of the environmental and genetic odds ratios, respectively). Six hundreds and seventeen individuals were then excluded due to missing genotyping data. The study then focused on the remaining group of 3,426 individuals with the required genetic and environmental data (Tables 1 and 2) and who did not leave the study with unknown T2D status. All of these individuals were free from diabetes at baseline and 402 developed T2D during the follow-up period. The 15-years incidence for diabetes was assessed.

**Table 1:** Distribution of individuals across environmental data characteristics.

Risk factor	Number of individuals (%)
Male	1,298 (37.9)
Female	2,128 (62.1)
Physical activity—Very low (MET-h/week)	3,238 (94.5)
Physical activity—Low (MET-h/week)	186 (5.4)
Physical activity—Moderate (MET-h/week)	2 (0.1)
Neither parent with T2D	3,337 (97.4)
One parent with T2D	89 (2.6)
Two parents with T2D	0 (0)
Never smoker	1,471 (42.9)
Past smoker	1,151 (33.6)
Current smoker—Sometimes	152 (4.44)
Current smoker—Regularly	652 (19.0)

MET, metabolic equivalent

**Table 2:** The environmental data characteristics in the MDC-CC.

Risk factor	Value (Mean ± SD)
Age (Baseline)	57.1 ± 5.93
BMI	25.6 ± 3.75
Alcohol (drinks/week)	5.15 ± 5.94
Systolic blood pressure (mmHg)	140 ± 18.3
Diastolic blood pressure (mmHg)	86.5 ± 9.24
Triglycerides (mmol/L)	1.32 ± 0.69
HDL-C (mmol/L)	1.41 ± 0.37

Diabetes cases were retrieved using record linkage of the Swedish personal Identification Code with six different national and regional diabetes registers: the Malmö HbA1c register (MHR) (see definition below), having a diagnosis of DM registered in the nationwide Swedish National Diabetes Register (NDR) [55] or the regional Diabetes 2000 register of the Scania region of which Malmö is the largest city [56], or the Swedish National Patient Register, which covers all somatic and psychiatric hospital discharges and Swedish Hospital-based outpatient care [57], or having diabetes as a cause of death in the Swedish Cause-of-Death Register [58], or having been prescribed anti-diabetic medication as registered in the Swedish Prescribed Drug Register [59].

The MHR analysed and catalogued all HbA1c samples at the Department of Clinical Chemistry taken in institutional and non-institutional care in the greater Malmö area from 1988 onwards. Individuals who had at least two HbA1c recordings ≥6.0% in the MHR using the Swedish Mono-S standardization system (corresponding to 7.0% according to the US National Glycohemoglobin Standardization Program [NGSP]) were considered as having diabetes.

In addition, diabetes at the baseline examination of MDC was obtained by self-report of a physician diagnosis or use of antidiabetic medication according to a questionnaire, or fasting whole blood glucose of ≥ 6.1 mmol/L (corresponding to fasting plasma glucose concentration of ≥7.0 mmol/L). Furthermore, a diabetes diagnosis could be captured at the MDC-CC re-investigation by self-report of a physician diagnosis or use of DM medication according to a questionnaire or fasting plasma glucose of ≥ 7.0 mmol/L or a 120-min value post OGTT plasma glucose > 11.0 mmol/L [60]. Finally, a diabetes diagnosis could be captured by fasting plasma glucose of ≥ 7.0 mmol/L which was analyzed in a re-investigation of about 1/3 of the MDC participants who also participated in the Malmö Preventive Project [61].

All participants provided written consent and the study was approved by Regional Ethical Review Board in Lund, Sweden.

### Genotyping

Genotyping of the MDC-CC was made using the HumanOmniExpressExomeBeadChip and iScan system (Illumina, San Diego, CA, USA) analyzing 850,000 common, low frequency and rare SNPs. 5,451 individuals were successfully genotyped, i.e. passed our QC criteria. Criteria for excluding SNPs and study participants are presented in S2 Table.

### Statistical analysis

The environmental and genetic (SNP) data for each individual was submitted to the RAE. The RAE engine identifies the OR for each risk factor based on the study used to represent that RF. An aggregate environmental OR was calculated for each individual by multiplying the ORs from individual environmental RFs. An aggregate genetic OR was calculated for each individual by multiplying the ORs from individual SNPs. In the models including both environmental and genetic factors, the aggregate odds ratio based on the environmental and genetic risk factors are multiplied together to get the final odds ratio.

The aggregate environmental and genetic odds ratios were used to divide the population into environmental and genetic risk groups. In deciding the number of environmental and genetic categories, we tested multiple combinations including all the combinations that split them into either two or three risk groups. While all combinations showed the same trend, the case of 2 environmental and 3 genetic categories most clearly demonstrated the results. The population was split into two equally sized environmental risk groups, named ERG1, ERG2, using the median environmental odds ratio in the cohort. Independently, the aggregate genetic odds ratios were used to divide the population into three equally sized genetic risk groups using tertiles of the genetic odds ratios in the population. The three subpopulations are named GRG1, GRG2 and GRG3. This categorization provides a total of six risk groups an individual can be assigned to. The analysis is performed on 10,000 samples (with replacement) from the original population. The result is used to calculate bootstrap confidence intervals and p-values for the measures reported in this study.

## Results

The characteristics of the 3,426 individuals from the MDC-CC used in this study are shown in Tables 1 and 2. Among the 21 environmental RFs and 154 genetic RFs that were utilized by the RAE for its T2D assessment, data for only 13 environmental and 139 genetic RFs was available from the MDC-CC (Tables 3 and 4). The 139 genetic RFs (SNPs) span 19 LD blocks in people of European descent. The RAE has a ranking system that scores SNPs in each LD block and selects the highest scoring SNP with available data in each LD block for use in the risk assessment. In 18 of the covered 19 LD blocks the data for the highest scoring SNP was available for the majority of the individuals. In one of the LD blocks the data for the highest scoring SNP was not available and thus the data for the second highest scoring SNP was picked by RAE. For an individual to be included in the analysis they must have data for SNPs in all 19 LD blocks. The list of SNPs used in the risk assessment are presented in Table 5.

**Table 3:** List of environmental RFs available in the RAE and the MDC-CC.

Risk Factor	RAE Data	MDC-CC Data
Age	Yes	Yes
Alcohol	Yes	Yes
BMI	Yes	Yes
Coffee consumption	Yes	No
Ethnicity	Yes	Yes
Family history	Yes	Yes
Gender	Yes	Yes
Gestational diabetes^a^	Yes	No
HDL	Yes	Yes
Hypertension*	Yes	Yes
Passive smoker	Yes	No
Past smoking	Yes	Yes
Physical activity	Yes	Yes
Polycystic ovary syndrome^a^	Yes	No
Processed meat	Yes	No
Red meat	Yes	No
Smoking	Yes	Yes
Soft drinks	Yes	No
Triglyceride	Yes	Yes
Vitamin D	Yes	No
Waist circumference	Yes	Yes

*Hypertension risk includes either elevated systolic or diastolic blood pressure

^a^ Female only RF

**Table 4:** Number of available environmental and genetic RFs in the RAE and the MDC-CC.

	RAE Data	MDC-CC Data
Environmental RFs	21	13
Genetic RFs	154	139

All genetic markers are single nucleotide polymorphisms (SNPs)

**Table 5:** The nineteen unique SNPs that were picked and used by RAE for T2D genetic risk assessment.

SNP	Gene	Chromosome	SNP Rank In The Underlying LD Block
rs17106184	FAF1	1	Highest Scoring
rs1020731	RBMS1	2	Highest Scoring
rs9502570	Intergenic	6	Highest Scoring
rs849135	JAZF1	7	Highest Scoring
rs1111875	Intergenic	10	Highest Scoring
rs243021	Intergenic	2	Highest Scoring
rs1801282	PPARG	3	Highest Scoring
rs4402960 ^a^	IGF2BP2	3	Highest Scoring
rs7756992	CDKAL1	6	Highest Scoring
rs7903146 ^b^	TCF7L2	10	Highest Scoring
rs1552224	ARAP1	11	Highest Scoring
rs1387153	Intergenic	11	Highest Scoring
rs4275659	ABCB9	12	Highest Scoring
rs702634	ARL15	5	Highest Scoring
rs231362	KCNQ1	11	Highest Scoring
rs9936385	FTO	16	Highest Scoring
rs972283^c^	Intergenic	7	Highest Scoring
rs10811661 ^c^	Intergenic	9	Highest Scoring
rs13266634 ^c^	SLC30A8	8	Second Highest Scoring

^a^ The second highest scoring SNP in this LD block (rs1470579) was used for 5 individuals in which genotyping failed for the highest scoring SNP.

^b^ The second highest scoring SNP in this LD block (rs7901695) was used for 8 individuals in which genotyping failed for the highest scoring SNP.

^c^ SNPs that showed an opposite odds ratio direction (increased risk vs. decreased risk) in the MDC-CC and the RAE.

### Evaluation of the environmental odds ratios

To evaluate the performance of the RAE’s environmental model, we calculated the AUC based only on the environmental ORs and compared it with the AUCs obtained with two well-known T2D scores, the Framingham score [4] and the Finnish score [5] for our population. The AUCs for each model are presented in Table 6. The AUC of the RAE is significantly higher than both the Framingham and Finnish scores (Bootstrap p-values 0.0017 and < 1 x 10^−4^, respectively).

**Table 6:** Comparison of the RAE’s environmental model with two well-known environmental risk scores for T2D.

Environmental Risk Score	AUC	P-value
RAE	0.72 (0.69, 0.74)	—
Framingham	0.69 (0.66, 0.71)	0.0017
Finnish	0.64 (0.61, 0.66)	< 1 x 10^−4^

### Comparison of SNP odds ratios

**Fig 4:**
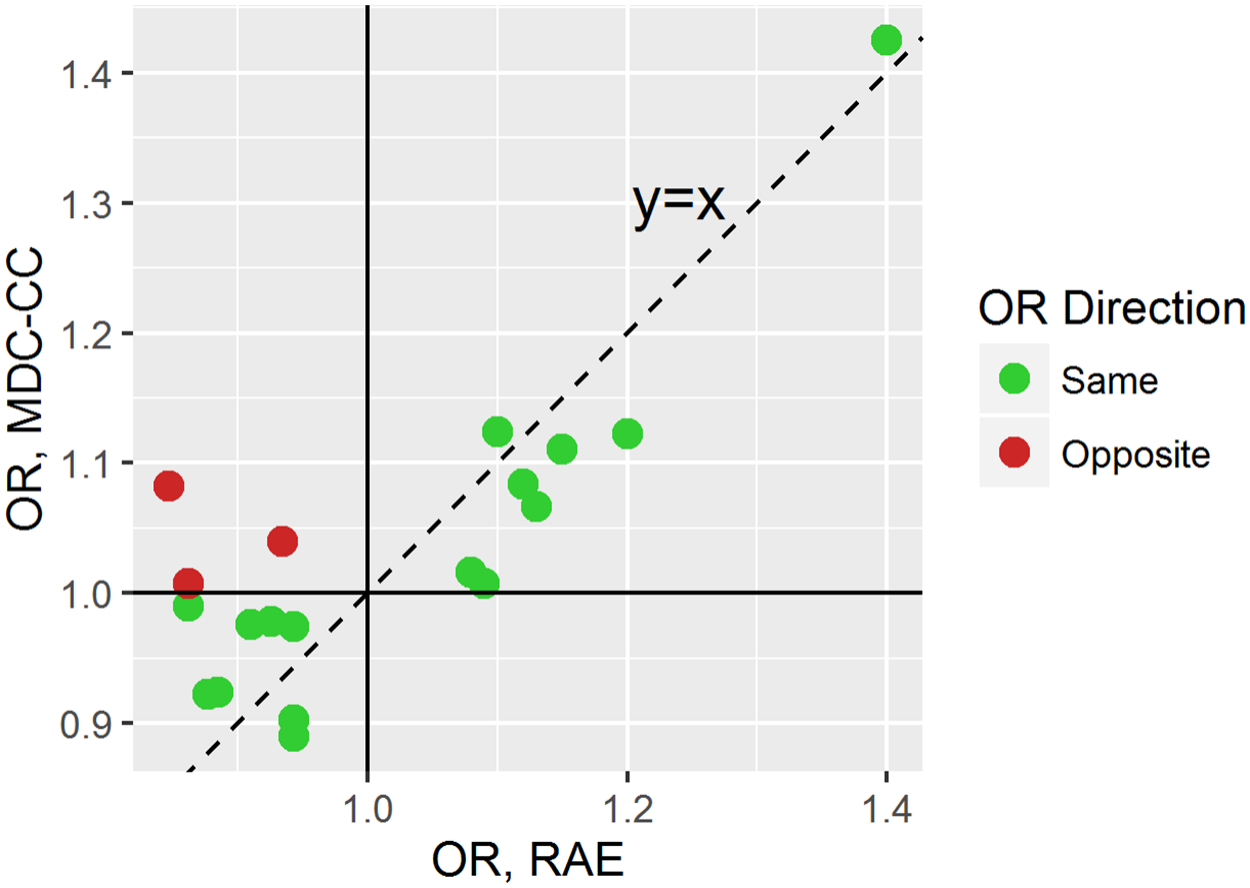
Relationship between the ORs for the 19 selected SNPs for type 2 diabetes in the RAE and the MDC-CC.

For the 19 representative SNPs used in the RAE’s genetic risk assessment, an OR was calculated from the MDC-CC for comparison. For the purpose of this comparison, we did not exclude MDC-CC individuals that had prevalent T2D at baseline or did not have sufficient environmental RF data in order to retain more T2D cases. The ORs for 16 SNPs (rs17106184, rs1020731, rs9502570, rs849135, rs1111875, rs243021, rs1801282, rs4402960, rs7756992, rs7903146, rs1552224, rs1387153, rs4275659, rs702634, rs231362, and rs9936385) were in the same direction in the RAE and the MDC-CC data. The mismatched OR effects for the other 3 SNPs (rs972283, rs10811661 and rs13266634) were only slightly different between the 2 groups (Fig 4). The ORs for rs972283 rs10811661 and rs13266634 were 1.04, 1.09 and 1.01 in the MDC-CC compared to 0.93, 0.85 and 0.86 in the RAE.

### Interaction between environmental and genetic risk

To measure the interaction between the genetic and environmental data, first two equally sized (n = 1,713) environmental risk groups (ERG1 and ERG2) were created using the median environmental odds ratio in the cohort. The median of the environmental ORs was 68.32, which is relatively large due to the effect of the age OR. The reference age for calculating the age OR is 20 years and all the individuals are at least 61 years old at the end of the follow-up period. Then, Pearson correlations between the genetic odds ratios and the case status of incident T2D were measured in ERG1 and ERG2 individually as well as in the full cohort. There were minor but significant correlations for all groups with coefficients of 0.086, 0.070, and 0.071 in ERG1 ([95% CI, 0.030–0.127], P-value = 2.8 x 10^−3^), ERG2 ([95% CI, 0.019–0.110], P-value = 7.8 x 10^−3^), and ERG1+ERG2 ([95% CI, 0.034–0.099], P-value = 2 x 10^−4^) respectively (Table 7).

**Table 7:** The correlation between genetic data and type 2 diabetes status in the MDC-CC.

Environmental Risk Group (ERG)	*r* (95% CI)	P-value (*r* ≠ 0)
ERG1 & ERG2	0.071 (0.034, 0.099)	2 × 10^−4^
ERG1	0.086 (0.030, 0.127)	2.8 × 10^−3^
ERG2	0.070 (0.019, 0.110)	7.8 × 10^−3^

ERG1 contains the individuals with total environmental ORs below the median environmental risk. ERG2 contains those above the median risk. ERG1 & ERG2 is the entire cohort.

**Fig 5:**
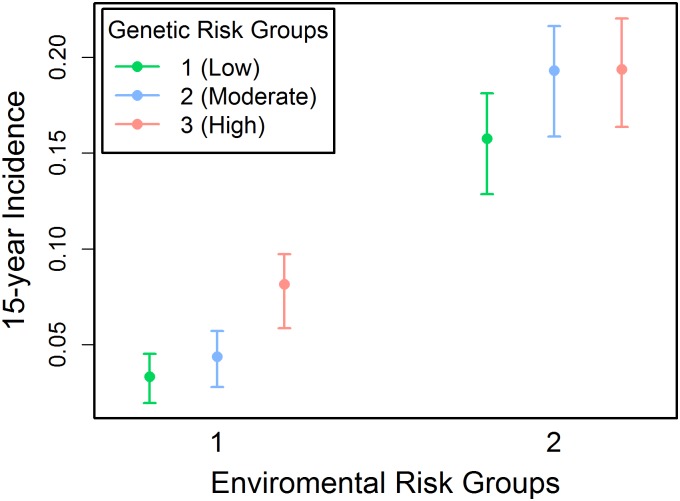
The type 2 diabetes 15-year incidence stratified by environmental and genetic risk groups in the MDC-CC. ERG1 contains the individuals with total environmental ORs below the median environmental risk. ERG2 contains those above the median risk. GRG1 has the individuals in the lowest tertile of total genetic risk, while GRG2 and GRG3 are the tertiles with moderate and high genetic risk individuals respectively.

Independently from the environmental risk groups, three equally sized (n = 1,142) genetic risk groups (GRG1, GRG2, and GRG3) were created based on the tertiles of the genetic odds ratios in the population. The genetic OR cutoffs used for this categorization were 0.755 and 1.049. This results in six combinations of environmental and genetic risk that each individual can be assigned to. Fig 5 plots 15-year incidence of T2D in each combination of risk groups. Within environmental risk group 1, the 15-year T2D incidence in genetic risk group 3 was 0.082 [95% CI, 0.059–0.097] compared to 0.033 [95% CI, 0.020–0.045] and 0.044 [95% CI, 0.028–0.057] in genetic risk groups 1 and 2 respectively (Table 8). When the same calculation was done for the individuals in environmental risk group 2, the 15-year incidences were almost the same in genetic risk group 2 (0.193 [95% CI, 0.159–0.216]) and 3 (0.194 [95% CI, 0.164–0.220]) and just slightly different from risk group 1 (0.158 [95% CI, 0.129–0.181]) (Table 8). Table 9 demonstrates p-values evaluating the significance of the difference in T2D incidences between each pair of genetic risk groups. The analysis is performed separately for each environmental risk group. As demonstrated in Table 9, the difference between the incidence of T2D in genetic risk group 3 and genetic risk groups 1 and 2 is significant only in the environmental risk group 1. This result implies the impact of genetic data on the incidence of type 2 diabetes might be different across different environmental risk groups, and thus the environmental risk level of an individual should be accounted for when evaluating the risk due to genetic factors.

**Table 8:** The 15-year type 2 diabetes incidence across different environmental and genetic risk groups.

	GRG 1 (95% CI)	GRG 2 (95% CI)	GRG 3 (95% CI)
**ERG 1**	0.033 (0.020, 0.045)	0.044 (0.028, 0.057)	0.082 (0.059, 0.097)
**ERG 2**	0.158 (0.129, 0.181)	0.193 (0.159, 0.216)	0.194 (0.164, 0.220)

Risk groups are the same as shown in Fig 5.

**Table 9:** P-values evaluating the significance of difference between 15-year incidences of T2D in different genetic risk groups.

	GRG 3 ≠ GRG 1	GRG 3 ≠ GRG 2	GRG 2 ≠ GRG 1
**ERG 1**	0.0011	0.0138	0.379
**ERG 2**	0.0952	0.841	0.154

Data is provided separately for environmental risk groups 1 and 2.

### Effect of genetic data on increasing the quality of T2D incidence prediction

As mentioned earlier, the RAE is used to get both an environmental and a genetic OR for each individual in the cohort. To assess the value of adding genetic data in increasing the quality of risk predictions based on an environmental model, the AUC was calculated for the environmental odds ratios alone and for the combined environmental and genetic odds ratios. The AUC for the environmental only model was 0.72 [95% CI, 0.69–0.74] and when the genetic data was added the AUC increased by 1% to 0.73 [95% CI, 0.70–0.75] (Table 10). This improvement was statistically significant (P-value = 0.042). We verified that practically the 1% improvement in the AUC will result in approximately 2% improvement in sensitivity or specificity of T2D incidence predictions in the MDC-CC.

**Table 10:** The AUC after running the RAE utilizing environmental data alone and combined with the genetic data.

Environmental Risk Group	AUC (Environmental Only)	AUC (Environmental + Genetic)	P-value
**ERG1 & ERG2**	0.72 (0.69, 0.74)	0.73 (0.70, 0.75)	0.042
**ERG1**	0.58 (0.52, 0.62)	0.63 (0.57, 0.67)	0.029
**ERG2**	0.65 (0.62, 0.68)	0.66 (0.63, 0.69)	0.248

Next, we studied how the value of genetic data in improving the quality of T2D predictions varies in different environmental risk groups. To this end, we performed the above analysis separately in ERG1 and ERG2. As shown in Table 10, there is a positive shift of 5% in AUCs in ERG1 and this shift is statistically significant (P-value = 0.029). There is also a positive shift of 1% in AUCs in ERG2 but the shift is not statistically significant (P-value = 0.248). This result supports the hypothesis that the value of genetic data is higher in populations with lower environmental risk levels.

The environmental factors used in this study did not include family history of diabetes. To compare the impact of genetic and family history data in improving the quality of risk assessment based only on environmental RFs, we calculated AUC in a model that consists only of environmental RFs and family history data (at the absence of genetic data). The resulting AUC was 0.72 [95% CI, 0.70–0.74]. The difference between this and the AUC obtained with the model including only environmental RFs is not significant (P-value = 0.333). This result supports the hypothesis that the genetic data has more value in increasing the quality of T2D predictions than family history data. A point to note, however, is that the prevalence of family history of T2D is low in the studied cohort (2.6% compared to previously reported values of ~20–40% [8, 16]). Further experiments are needed to compare the effects of genetic and family history data in improving the prediction of T2D.

## Discussion

Our objective with this study was to measure the effect of utilizing both genetic and environmental data to predict the incidence of type 2 diabetes in a Swedish cohort. Frequently, studies of human health and common complex diseases have focused on identifying either genetic or environmental RFs that could explain variation in disease susceptibility. Type 2 diabetes is a multifactorial disease caused by both genetic and environmental RFs. Therefore, it’s important to have the ability to accurately measure the impact of each RF individually and in combination with other RFs.

We aimed to apply the risk assessment engine to evaluate the T2D risk of individuals in the MDC-CC. We found that 62% of the environmental factors that are used by the RAE were available in the data for the MDC-CC. The environmental model used by the RAE performed significantly better than two existing T2D risk scores (Framingham and Finnish). Of the 24 LD blocks typically used by the RAE for the T2D genetic risk assessment, data for 19 LD blocks was available for the MDC-CC. Among the 19 representative SNPs that were used for the analysis, 16 of them showed the same odds ratio direction (increased risk/decreased risk) for T2D in the MDC-CC as in the RAE. The 3 SNPs whose ORs’ direction didn’t match (increased risk /decreased risk) had ORs of 1.04, 1.09 and 1.01 in MDC-CC compared to 0.93, 0.85 and 0.86 in the RAE. This slight difference in ORs for these SNPs between the two data sets could be due to the specific ethnicities studied in each dataset or just due to the random nature of the studies.

To study the interaction between genetic and environmental RFs, the MDC-CC was divided into two environmental risk groups. It was demonstrated that there is a positive correlation between genetic risk predisposition and incidence of type 2 diabetes in the whole population as well as in each of the two environmental risk groups. This implies the correlation between the genetic risk predisposition and incidence of type 2 diabetes holds regardless of the non-genetic risk group of the individual.

Independent from environmental risk groups, the population was divided into three genetic risk groups. After measuring the incidence of T2D in each of the risk categories, the effect of genetics on type 2 diabetes is more pronounced in individuals who do not carry many environmental RFs as compared to those who are at higher risk due to environmental factors. This indicates that genotyping individuals who are already at high-risk for type 2 diabetes due to their environmental RFs is of lesser value for their T2D risk stratification. Furthermore, it was demonstrated that even those phenotypically high-risk individuals who have the lowest possible genetic risk will still have higher type 2 diabetes risk than individuals who have high genetic risk but few environmental RFs. This finding is in concordance with previous findings about the effect of environmental RFs on the appearance of type 2 diabetes in various populations and how reduction of these RFs can minimize the total risk of developing T2D [6–7].

In order to evaluate whether the addition of genetic data increases the quality of predictions beyond what is achievable by environmental RFs, AUCs were calculated in the presence and absence of genetic odds ratios. It was demonstrated that adding genetic data results in a minor but significant positive shift in the AUC. The resulting AUCs were 0.72 [95% CI, 0.69–0.74] for the model including environmental RFs only and 0.73 [95% CI, 0.70–0.75] for the model including both genetic and environmental RFs. Talmud et al. [62] studied the effect of adding genetic data to an environmental risk prediction by combining a 65 SNP genetic risk score and the Framingham Offspring diabetes risk score. The combined risk score improved the AUC by 1% over the Framingham risk score alone (from 0.75 to 0.76), which is the same we saw when adding the genetic model to the RAE despite using fewer SNPs. Although their phenotypic and combined AUCs were slightly higher, when we tested the Framingham risk score on our population it performed significantly worse than the RAE’s environmental risk model.

Two factors in our study make this prediction problem challenging. One factor is the time horizon of the study is relatively long (15-years) and the other factor is that all participants are relatively old, between 61–83 years old at the endpoint. To compare the effect of genetic data with family history data, we repeated the above experiment with the genetic data being replaced with the family history data. We noticed the increase in the AUC with family history data is no longer significant. This result supports the hypothesis that genetic data is more valuable than family history data in improving the T2D incidence prediction. A point to note is that the prevalence of family history of T2D is low in the studied cohort (2.6% compared to previously reported values of ~20–40% [8,16]). The low prevalence of family history among the diabetic patients in the MDC-CC may be due to some missing family history data in the cohort. Further experiments are needed to establish the relative values of genetic and family history data in improving the prediction of T2D.

To study the value of genetic data in improving T2D prediction in different environmental risk groups, we studied the additional value of genetic data separately in risk categories ERG1 and ERG2. Specifically, we calculated AUCs of a model based on environmental RFs at the presence and absence of genetic data separately in each of the risk categories ERG1 and ERG2. We demonstrated that the increase in the AUC is statistically significant only in the ERG1. This finding again supports the hypothesis that the value of genetic data in improving the prediction of incident T2D is higher in populations that are at lower environmental risk for T2D. The values of AUC are lower in each of the risk categories ERG1 and ERG2 compared to the whole population. That is because, by design, individuals in each of these risk categories are similar in terms of their environmental risk scores making it difficult to use their environmental risk scores to determine their risk for a certain condition.

In conclusion, the results of this study demonstrate that inclusion of genetic data in a comprehensive risk assessment engine can make a slight improvement in predicting type 2 diabetes incidence compared to environmental factors alone. We believe addition of new reliable genetic markers that will be discovered in future might increase the level of association between genotypic and phenotypic data for T2D. In this study, the genetic data brought higher value in individuals with a lower environmental risk. Even those individuals who have genetic predisposition for the disease can reduce their total disease risk by managing environmental RFs. Although genetic predisposition for a disease is something that cannot be modified, the power of knowing that information can help the individual be more thoughtful about managing and controlling the associated environmental RFs. The recognition that environmental factors that increase risk for type 2 diabetes may be also driven by genetic factors and modified by environmental factors patterns a rich yet complex paradigm for designing additional testing intervention strategies in the future.

## Supporting information

S1 File PubMed searches used for the environmental RFs. (DOCX)

S1 Table Odds ratios (or OR ranges) used by the RAE’s environmental and genetic risk models in this study. **A)** Odds ratio ranges for the 21 environmental risk factors used in the study. B) Allelic odds ratios for the SNPs used by the RAE. * SNPs are not available on the OmniExpress array and thus could not be used in the analysis.
(DOCX)

S2 Table Quality control (QC) criteria in genotyping of MDC-CC on marker and individual levels. Variants or individuals were excluded if they met any of these criteria.
(DOCX)
