# Supplementary material for: Utilization of genetic data can improve the prediction of type 2 diabetes incidence in a Swedish cohort
Source: PLoS One. 2017 Jul 12;12(7):e0180180. doi: 10.1371/journal.pone.0180180 (PMC5507496; doi:10.1371/journal.pone.0180180)
Supplement: S1 File — (DOCX) [file pone.0180180.s001.docx]

"Diabetes Mellitus, Type 2"[Mesh] AND "smoking"[Mesh] AND ("2000/01/01"[PDAT] : "2015/07/31"[PDAT]) AND "humans"[MeSH Terms] AND English[lang]

"Diabetes Mellitus, Type 2"[Mesh] AND "Coffee"[Mesh] AND (("2000/01/01"[PDAT] : "2015/07/31"[PDAT]) AND "humans"[MeSH Terms] AND English[lang])

"Diabetes Mellitus, Type 2/prevention and control"[MAJR]

"Diabetes Mellitus, Type 2"[Mesh] AND "Body Mass Index"[Mesh] AND ("2000/01/01"[PDAT] : "2015/07/31"[PDAT]) AND "humans"[MeSH Terms] AND English[lang]

"Diabetes Mellitus, Type 2"[Mesh] AND "Obesity, Abdominal"[Mesh] AND (("2000/01/01"[PDAT] : "2015/07/31"[PDAT]) AND "humans"[MeSH Terms] AND English[lang]) "Diabetes Mellitus, Type 2/prevention and control"[MAJR]

"Diabetes Mellitus, Type 2"[Mesh] AND "Obesity"[Mesh] AND (("2000/01/01"[PDAT] : "2015/07/31"[PDAT])) AND "humans"[MeSH Terms] AND English[lang]

"Diabetes Mellitus, Type 2"[Mesh] AND "Coffee"[Mesh] AND (("2000/01/01"[PDAT] : "2015/07/31"[PDAT]) AND "humans"[MeSH Terms] AND English[lang])

"Diabetes Mellitus, Type 2/prevention and control"[MAJR]

"Diabetes Mellitus, Type 2"[Mesh] AND "Ethanol"[Mesh] AND ("2000/01/01"[PDAT] : "2015/07/31"[PDAT]) AND "humans"[MeSH Terms] AND English[lang]

"Diabetes Mellitus, Type 2"[Mesh] AND "Blood Pressure"[Mesh] AND ("2000/01/01"[PDAT] : "2015/07/31"[PDAT]) AND "humans"[MeSH Terms] AND English[lang]

"Diabetes Mellitus, Type 2"[Mesh] AND "Meat Products"[Mesh] AND ("2000/01/01"[PDAT] : "2015/07/31"[PDAT]) AND "humans"[MeSH Terms] AND English[lang]

"Diabetes Mellitus, Type 2"[Mesh] red meat AND ("2000/01/01"[PDAT] : "2015/07/31"[PDAT]) AND "humans"[MeSH Terms] AND English[lang]

"Diabetes Mellitus, Type 2"[Mesh] AND "Gender Identity"[Mesh] AND (("2000/01/01"[PDAT] : "2015/07/31"[PDAT])) AND "humans"[MeSH Terms] AND English[lang]

"Diabetes Mellitus, Type 2"[Mesh] family history AND (("2000/01/01"[PDAT] : "2015/07/31"[PDAT])) AND "humans"[MeSH Terms] AND English[lang]

"Diabetes Mellitus, Type 2"[Mesh] AND "Prediabetic State"[Mesh] AND ("2000/01/01"[PDAT] : "2015/07/31"[PDAT]) AND "humans"[MeSH Terms] AND English[lang]

"Diabetes Mellitus, Type 2"[Mesh] AND "Ethanol"[Mesh] AND (("2000/01/01"[PDAT] : "2015/07/31"[PDAT])) AND "humans"[MeSH Terms] AND English[lang]

"Diabetes Mellitus, Type 2"[Mesh] AND "Exercise"[Mesh] AND (("2000/01/01"[PDAT] : "2015/07/31"[PDAT])) AND "humans"[MeSH Terms] AND English[lang]

"Diabetes Mellitus, Type 2"[Mesh] AND "Polycystic Ovary Syndrome"[Mesh] AND (("2000/01/01"[PDAT] : "2015/07/31"[PDAT])) AND "humans"[MeSH Terms] AND English[lang])

"Diabetes Mellitus, Type 2"[Mesh] soft drink AND (("2000/01/01"[PDAT] : "2015/07/31"[PDAT])) AND "humans"[MeSH Terms] AND English[lang]

"Diabetes Mellitus, Type 2"[Mesh] AND "Sleep Apnea Syndromes"[Mesh] AND ("2000/01/01"[PDAT] : "2015/07/31"[PDAT]) AND "humans"[MeSH Terms] AND English[lang]

"Diabetes Mellitus, Type 2"[Mesh] soda AND (("2000/01/01"[PDAT] : "2015/07/31"[PDAT])) AND "humans"[MeSH Terms] AND English[lang]

"Diabetes Mellitus, Type 2"[Mesh] AND "Vitamin D"[Mesh] AND ("2000/01/01"[PDAT] : "2015/07/31"[PDAT]) AND "humans"[MeSH Terms] AND English[lang]

"Diabetes Mellitus, Type 2"[Mesh] AND "Waist Circumference"[Mesh] AND (("2000/01/01"[PDAT] : "2015/07/31"[PDAT])) AND "humans"[MeSH Terms] AND English[lang]
