# Supplementary material for: Utilization of genetic data can improve the prediction of type 2 diabetes incidence in a Swedish cohort
Source: PLoS One. 2017 Jul 12;12(7):e0180180. doi: 10.1371/journal.pone.0180180 (PMC5507496; doi:10.1371/journal.pone.0180180)
Supplement: S1 Table — A) Odds ratio ranges for the 21 environmental risk factors used in the study. B) Allelic odds ratios for the SNPs used by the RAE. * SNPs are not available on the OmniExpress array and thus could not be used in the analysis. (DOCX) [file pone.0180180.s002.docx]

(A)

|  | **Risk Factor** | **OR Range** |
| --- | --- | --- |
| 1 | Age | 1-22.2 |
| 2 | Alcohol | 0.55-1 (female); 0.71-1 (male) |
| 3 | BMI | 1-11.58 |
| 4 | Coffee consumption | 0.61-1 |
| 5 | Ethnicity | 1-3 |
| 6 | Family history | 1-6.1 |
| 7 | Gender | 1-1.56 |
| 8 | Gestational diabetes | 1-7.43 |
| 9 | HDL | 1-2.55 |
| 10 | Hypertension | 0.66-1.96 |
| 11 | Passive smoker | 1-1.16 |
| 12 | Past smoking | 1-1.28 |
| 13 | Physical activity | 0.57-1 |
| 14 | Polycystic ovary syndrome | 1-4.43 |
| 15 | Processed meat | 1-1.16 |
| 16 | Red meat | 1-1.2 |
| 17 | Smoking | 1-1.98 |
| 18 | Soft drinks | 1-1.24 |
| 19 | Triglyceride | 1-2.11 |
| 20 | Vitamin D | 1-3.32 |
| 21 | Waist circumference | 0.61-1.47 |

(B)

|  | **SNP** | **OR (Increased risk allele)** | **Used in the Analysis** |
| --- | --- | --- | --- |
| 1 | rs17106184 | 1.1 | Yes |
| 2 | rs1020731 | 1/0.91 | Yes |
| 3 | rs9502570 | 1.06 | Yes |
| 4 | rs849135 | 1.12 | Yes |
| 5 | rs1111875 | 1.15 | Yes |
| 6 | rs243021 | 1.08 | Yes |
| 7 | rs1801282 | 1.16 | Yes |
| 8 | rs4402960 | 1.13 | Yes |
| 9 | rs1470579 | 1/0.940 | Yes (Backup to rs4402960) |
| 10 | rs7756992 | 1.2 | Yes |
| 11 | rs7903146 | 1.4 | Yes |
| 12 | rs7901695 | 1.660 | Yes (Backup to rs7903146) |
| 13 | rs1552224 | 1.14 | Yes |
| 14 | rs1387153 | 1.09 | Yes |
| 15 | rs4275659 | 1.06 | Yes |
| 16 | rs702634 | 1.06 | Yes |
| 17 | rs231362 | 1.08 | Yes |
| 18 | rs9936385 | 1.13 | Yes |
| 19 | rs972283 | 1.07 | Yes |
| 20 | rs10811661 | 1.18 | Yes |
| 21 | rs13266634 | 1.16 | Yes |
| 22 | rs6813195 | 1.08 | No* |
| 23 | rs3132524 | 1.07 | No* |
| 24 | rs7578326 | 1.11 | No* |
| 25 | rs896854 | 1.06 | No* |
| 26 | rs13292136 | 1.11 | No* |
