# Supplementary material for: Utilization of genetic data can improve the prediction of type 2 diabetes incidence in a Swedish cohort
Source: PLoS One. 2017 Jul 12;12(7):e0180180. doi: 10.1371/journal.pone.0180180 (PMC5507496; doi:10.1371/journal.pone.0180180)
Supplement: S2 Table — Variants or individuals were excluded if they met any of these criteria. (DOCX) [file pone.0180180.s003.docx]

| Marker Level | Individual Level |
| --- | --- |
| Call rate <95% | Call rate <95% |
| Variants on sex chromosomes | Discordant sex in self-report vs genetically determined sex |
| Variants on mitochondrial DNA | A second degree relatedness or higher within the sample, based on identity by descent sharing calculations |
| Variants showing an extreme deviation from Hardy-Weinberg equilibrium (P-value < 1 × 10^-6^). | Population outliers based on inspection of the first two principal component plots. |
|  | Inbreeding coefficient 0.2 or higher |
